# The heme-regulated inhibitor kinase requires dimerization for heme-sensing activity

**DOI:** 10.1016/j.jbc.2022.102451

**Published:** 2022-09-03

**Authors:** M. Daniel Ricketts, Ryan P. Emptage, Gerd A. Blobel, Ronen Marmorstein

**Affiliations:** 1 Department of Biochemistry and Biophysics and the Abramson Family Cancer Research Institute, Perelman School of Medicine, University of Pennsylvania, Philadelphia, Pennsylvania, USA; 2 Division of Hematology, Children’s Hospital of Philadelphia, Perelman School of Medicine, University of Pennsylvania, Philadelphia, Pennsylvania, USA

**Keywords:** heme-regulated inhibitor, eIF2α phosphorylation, heme binding, heme regulation, β-hemoglobinopathies, β-thalassemia, globin translation, biophysics, protein dimerization, DSF, differential scanning fluorimetry, eIF2α, eukaryotic initiation factor 2α, HbF, fetal hemoglobin, HRI, heme-regulated inhibitor, HRI-FL, full-length HRI, MW, molecular weight, PKR, protein kinase R, TCEP, tris(2-carboxyethyl)phosphine

## Abstract

The heme-regulated inhibitor (HRI) is a heme-sensing kinase that regulates mRNA translation in erythroid cells. In heme deficiency, HRI is activated to phosphorylate eukaryotic initiation factor 2α and halt production of globins, thus avoiding accumulation of heme-free globin chains. HRI is inhibited by heme *via* binding to one or two heme-binding domains within the HRI N-terminal and kinase domains. HRI has recently been found to inhibit fetal hemoglobin (HbF) production in adult erythroid cells. Depletion of HRI increases HbF production, presenting a therapeutically exploitable target for the treatment of patients with sickle cell disease or thalassemia, which benefit from elevated HbF levels. HRI is known to be an oligomeric enzyme that is activated through autophosphorylation, although the exact nature of the HRI oligomer, its relation to autophosphorylation, and its mode of heme regulation remain unclear. Here, we employ biochemical and biophysical studies to demonstrate that HRI forms a dimeric species that is not dependent on autophosphorylation, the C-terminal coiled-coil domain in HRI is essential for dimer formation, and dimer formation facilitates efficient autophosphorylation and activation of HRI. We also employ kinetic studies to demonstrate that the primary avenue by which heme inhibits HRI is through the heme-binding site within the kinase domain, and that this inhibition is relatively independent of binding of ATP and eukaryotic initiation factor 2α substrates. Together, these studies highlight the mode of heme inhibition and the importance of dimerization in human HRI heme-sensing activity.

A family of four stress kinases comprised of protein kinase R (PKR)–like endoplasmic reticulum kinase, PKR, general control nondepressible 2, and heme-regulated inhibitor (HRI), phosphorylate serine 51 in eukaryotic initiation factor 2α (eIF2α) in order to terminate protein translation in response to cellular stress (1). PKR-like endoplasmic reticulum kinase is activated upon an accumulation of misfolded proteins in the endoplasmic reticulum as part of the unfolded protein response, PKR is primarily activated upon binding to dsRNA, and general control nondepressible 2 is activated upon amino acid deficiency through binding to uncharged tRNAs. HRI kinase is activated upon heme deficiency, although it also activated in response to several additional stimuli, such as exposure to heat shock, oxidative stress, proteasome inhibition, inhibition of Hsc70 binding, and mitochondrial damage (1, 2, 3, 4, 5).

HRI is a key regulator of globin synthesis and erythropoiesis (4, 6, 7, 8, 9). HRI functions as a heme sensor, modulating globin synthesis with the amount of heme available for hemoglobin production (1, 6, 8). If heme levels are sufficiently high, HRI is inhibited and globin synthesis proceeds; in heme-deficient conditions, HRI is activated to phosphorylate eIF2α, terminating globin translation (6, 9). In HRI-depleted erythroid cells, excessive globins build up during heme-deficient conditions and form inclusion bodies, leading to proteotoxicity (7, 8). Recently, depletion of HRI has been shown to activate production of fetal hemoglobin (HbF) (10, 11). Reactivation of HbF can alleviate the severity of β-hemoglobinopathies such as β-thalassemia and sickle cell disease (10, 11). This relationship between HRI activity and HbF production has led to the emergence of HRI as a potential therapeutic target for β-hemoglobinopathies (12).

HRI is composed of an N-terminal heme-binding domain, a kinase domain harboring a putatively unstructured kinase-insert region within the N-terminal lobe, an unusually large activation loop, and a C-terminal coiled-coil domain (Fig. 1). HRI has been previously reported to form an oligomer that is heavily autophosphorylated at as many as 33 amino acid sites, at least some of which facilitate HRI autophosphorylation and eIF2α phosphorylation activity (13, 14, 15, 16, 17, 18). HRI has two proposed heme-binding regions, one in the N-terminal domain, and a second within the kinase domain (14, 19). HRI is inhibited through heme association with one or both the heme-binding domains (19), and heme dissociation allows for HRI autophosphorylation and activation (13, 19).

**Figure 1:**
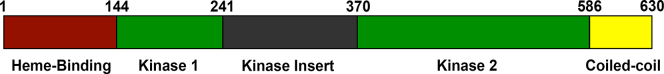
**HRI domain architecture.** HRI domain map illustrating delineations between key HRI functional domains. HRI, heme-regulated inhibitor.

Here, we report that HRI forms a stable dimer in solution. HRI dimer formation is mediated through the C-terminal coiled-coil domain, deletion of which leads to a monomeric species in solution. We also show that HRI dimer formation facilitates efficient autophosphorylation but is independent of the autophosphorylation state of HRI. This suggests that HRI has a mechanism of transactivation, in which one molecule within the dimer phosphorylates the adjacent molecule, leading to HRI activation. Finally, we show that hemin inhibition of HRI is relatively independent of binding of both ATP and an eIF2α substrate peptide and is mediated predominantly by the HRI kinase domain.

## Results

### HRI purifies as an active autophosphorylated dimer

**Figure 2:**
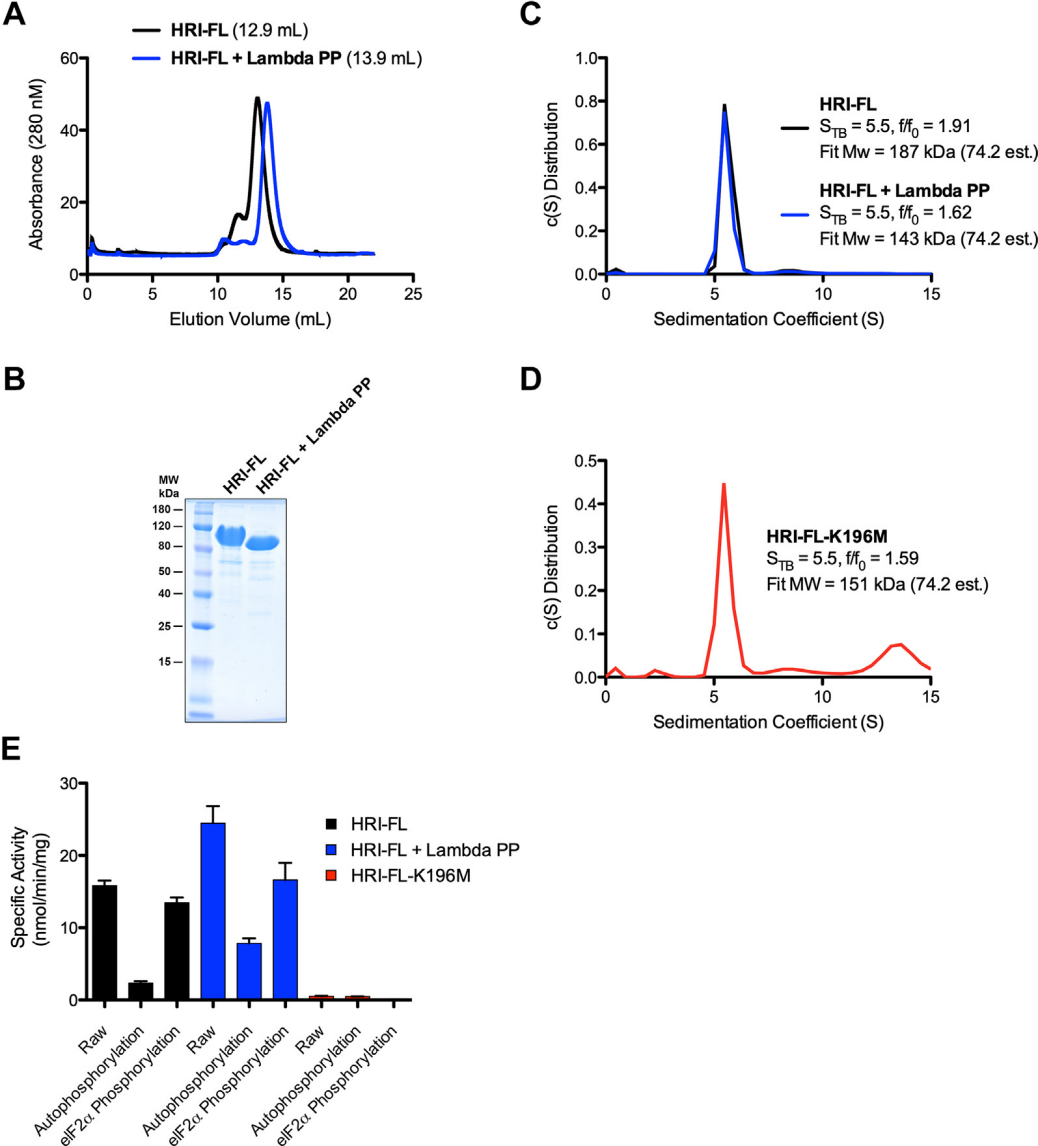
**HRI purification, phosphorylation status, and multimerization properties.***A*, HRI-FL and lambda phosphatase treated HRI-FL elute at different volumes from a Superdex 200 size-exclusion column. *B*, treatment of HRI-FL with lambda phosphatase results in a visible decrease in MW on an SDS-PAGE gel. *C*, sedimentation velocity analysis of HRI-FL and lambda phosphatase–treated HRI-FL. *D*, sedimentation velocity analysis of HRI-FL-K196M. *E*, analysis of eIF2α peptide phosphorylation activity and autophosphorylation activity of HRI-FL, lambda phosphatase–treated HRI-FL, and HRI-FL-K196M, N = 2. eIF2α, eukaryotic initiation factor 2α; HRI, heme-regulated inhibitor; HRI-FL, full-length HRI; MW, molecular weight.

Full-length HRI (HRI-FL) purified recombinantly from *Escherichia coli* runs as a single monodisperse peak at 12.9 ml on a S200 size-exclusion column (Fig. 2*A*). Treatment with lambda phosphatase results in a 1 ml shift of the primary peak to 13.9 ml (Fig. 2*A*). Mouse HRI has been reported to have as many as 33 autophosphorylation sites (13). The total mass of 33 phosphate groups is approximately 3.1 KDa. Comparison of HRI-FL and dephosphorylated HRI-FL on an SDS-PAGE gel reveals a small decrease in molecular weight (MW) for the dephosphorylated sample, which may represent the loss of ∼3 KDa. This difference indicates that HRI-FL purified from *E. coli* cells is heavily autophosphorylated and that lambda phosphatase is able to remove a significant number of the phosphate groups (Fig. 2*B*). As it is unlikely that such a small decrease in mass would alone be responsible for such a large shift in the size-exclusion elution profile, this large shift may indicate a change in oligomerization state or a structural rearrangement to a more globular conformation upon dephosphorylation.

To further dissect the conformational and/or oligomerization change that occurs upon dephosphorylation, sedimentation velocity analysis was performed on HRI-FL and dephosphorylated HRI-FL (Fig. 2*C*). A single 5.5 S_T,B_ species was observed for both samples, although both proteins had different sedimentation coefficients: HRI-FL had an *f*/*f*_0_ = 1.91, whereas dephosphorylated HRI-FL had an *f*/*f*_0_ = 1.62. The higher frictional ratio for HRI-FL indicates a more elongated species in solution, in comparison to a more compact species for dephosphorylated HRI-FL (20, 21). HRI-FL fit to an MW of 187 kDa, and dephosphorylated HRI-FL fit to an MW of 143 kDa. Dimers of phosphorylated HRI or dephosphorylated HRI have theoretical MWs of 154.6 and 148.4 kDa, respectively. The fit MW for both HRI species indicates the presence of stable dimers in solution regardless of phosphorylation state. A catalytically dead mutant, HRI-FL-K196M, was also purified and subjected to sedimentation velocity. A single 5.5 S_T,B_ species with an *f*/*f*_0_ = 1.59 was observed with a fit MW of 151 kDa (Fig. 2*D*), indicating that the HRI dimer is stable in the absence of autophosphorylation.

It has been reported that autophosphorylation is essential for HRI enzymatic activity (17, 18). To decipher the relationship between HRI phosphorylation state and enzymatic activity, HRI-FL and dephosphorylated HRI-FL were compared for eIF2α peptide phosphorylation activity. To do this, we employed a P^32^ ATP assay that captures both phosphorylated HRI, and the eIF2α peptide substrate modified P^32^ on negatively charged P81 filter paper. To analyze peptide phosphorylation, a reaction lacking peptide was carried out to quantify the autophosphorylation background. The autophosphorylation background was subtracted from the raw signal of the reaction containing both HRI and eIF2α peptide to yield the signal from peptide phosphorylation. Comparison of HRI-FL and dephosphorylated HRI-FL reveals that both have comparable activity toward the eIF2α peptide, whereas the dephosphorylated HRI has significantly higher autophosphorylation signal (Fig. 2*E*). As a negative control, a catalytically defective HRI-FL-K196M mutant showed no detectable radioactive signal. Together, these data suggest that dephosphorylated HRI is able to rapidly autophosphorylate and maintain efficient eIF2α peptide phosphorylation activity.

### Hemin does not directly compete with ATP or the eIF2α peptide to inhibit HRI

**Figure 3:**
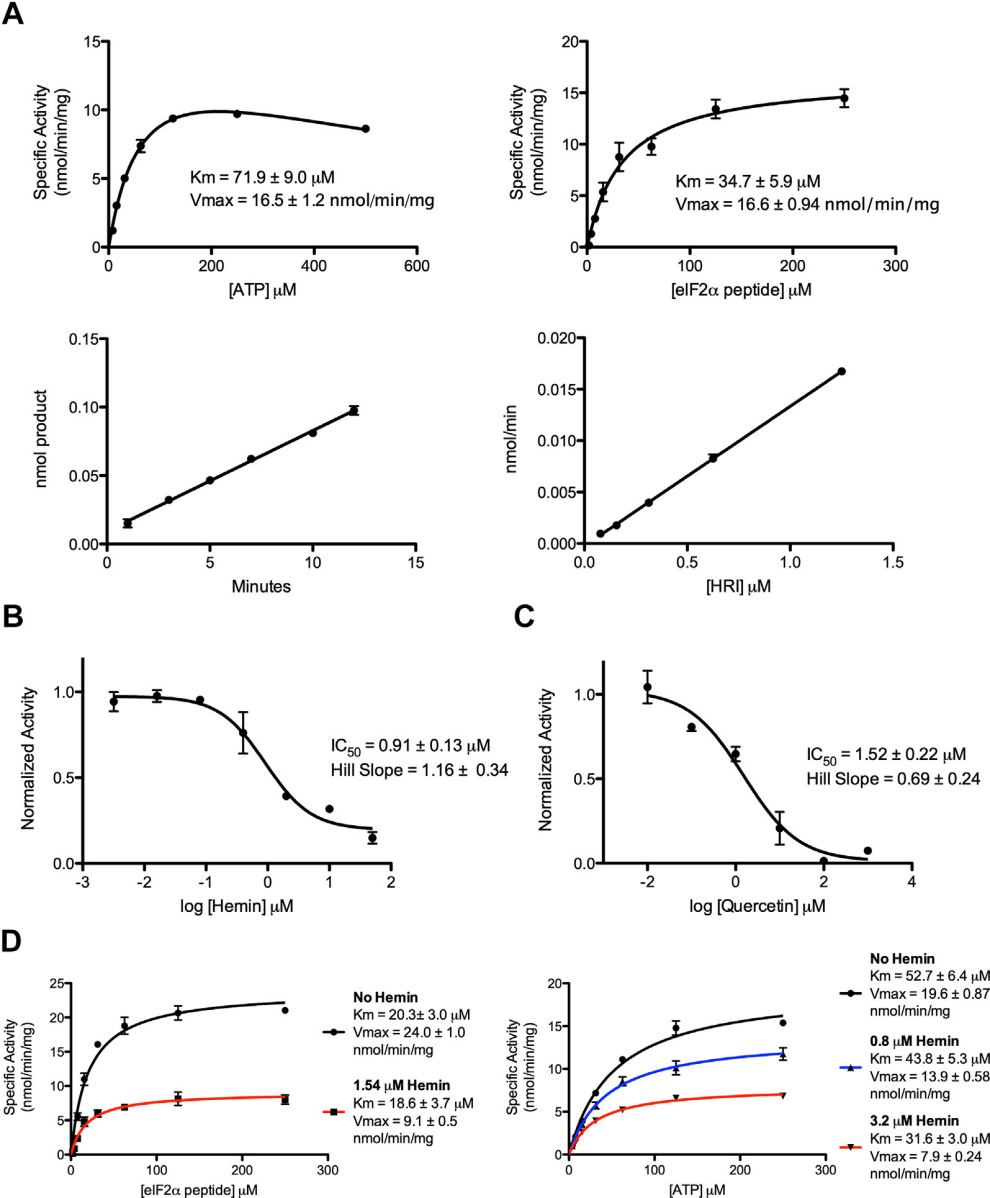
**Kinetic analysis of HRI-FL.***A*, steady-state kinetic analysis of HRI-FL. *B*, IC_50_ determination for hemin. *C*, IC_50_ determination for quercetin. *D*, analysis of mode of inhibition for hemin. HRI, heme-regulated inhibitor; HRI-FL, full-length HRI.

HRI phosphorylation of eIF2α peptide was subject to steady-state kinetic analysis to determine optimal kinase assay parameters. The signal for HRI autophosphorylation was subtracted from the total phosphorylation signal of each data point to yield the most quantitative analysis of peptide phosphorylation activity. Time and enzyme concentration were varied to ensure the time point for kinetic analysis, and [HRI] were within the linear range of product formation and rate (Fig. 3*A*, *bottom*). Michaelis–Menten curves were generated for both ATP and eIF2α peptide (Fig. 3*A*, *top*). This analysis revealed that HRI-FL has a *K*_*m*_ of 34.7 μM for the eIF2α peptide, with *V*_max_ of 16.6 nmol/min/mg. HRI was observed to have a *K*_*m*_ of 71.9 μM for ATP, with substrate inhibition at higher concentrations. The ATP curve was fit with the substrate inhibition model in GraphPad Prism (GraphPad Software, Inc) to determine a *V*_max_ of 16.5 nmol/min/mg.

Inhibition of HRI by heme binding has been widely reported (13, 14, 15, 16, 17, 18, 19). In addition, it has been reported that HRI is inhibited by quercetin, a pan-kinase ATP-competitive inhibitor (22, 23). IC_50_ curves were generated for HRI in the presence of both hemin and quercetin (Fig. 3, *B* and *C*). Hemin was determined to have an IC_50_ of 0.91 μM, whereas quercetin was determined to have an IC_50_ of 1.52 μM, at protein, ATP, and peptide concentrations of 0.5, 100, and 100 μM, respectively. Both values are in close agreement with previously published data (13, 15, 23). Notably, Hill coefficients close to one indicate that both molecules bind without apparent cooperativity.

While quercetin is known to be ATP competitive, the mode of inhibition for heme is less clear. It has been previously reported that HRI has two heme-binding sites, one in the N-terminal domain and one in the kinase domain (14, 15, 16, 19, 24). Mutagenesis studies indicate that the kinase domain heme-binding site is not close to the active site of HRI, suggesting that heme inhibition is likely noncompetitive with eIF2α and ATP (19). We conducted Michaelis–Menten analysis of HRI-FL for both ATP and eIF2α peptide in the presence of hemin. While the *V*_max_ decreased upon addition of hemin for peptide titrated in the presence of saturating ATP, the *K*_*m*_ values in the presence of hemin were very comparable to the *K*_*m*_ values without hemin; indicating hemin is not directly competing with the eIF2α substrate peptide (Fig. 3*D*, *first panel*). Similar analysis for ATP titrated in the presence of saturating eIF2α peptide indicated similar but drifting *K*_*m*_ values at increasing hemin. Given the three datasets generated for the ATP titration, we subjected the ATP titrations to a global fit for competitive as well as noncompetitive inhibition to directly compare these modes. The fit for noncompetitive inhibition (Fig. 3*D*, *second panel*) was clearly better than the fit for competitive inhibition based on R-squared and sum of squares analysis (data not shown).

### Phosphorylation and hemin binding does not stabilize HRI

**Figure 4:**
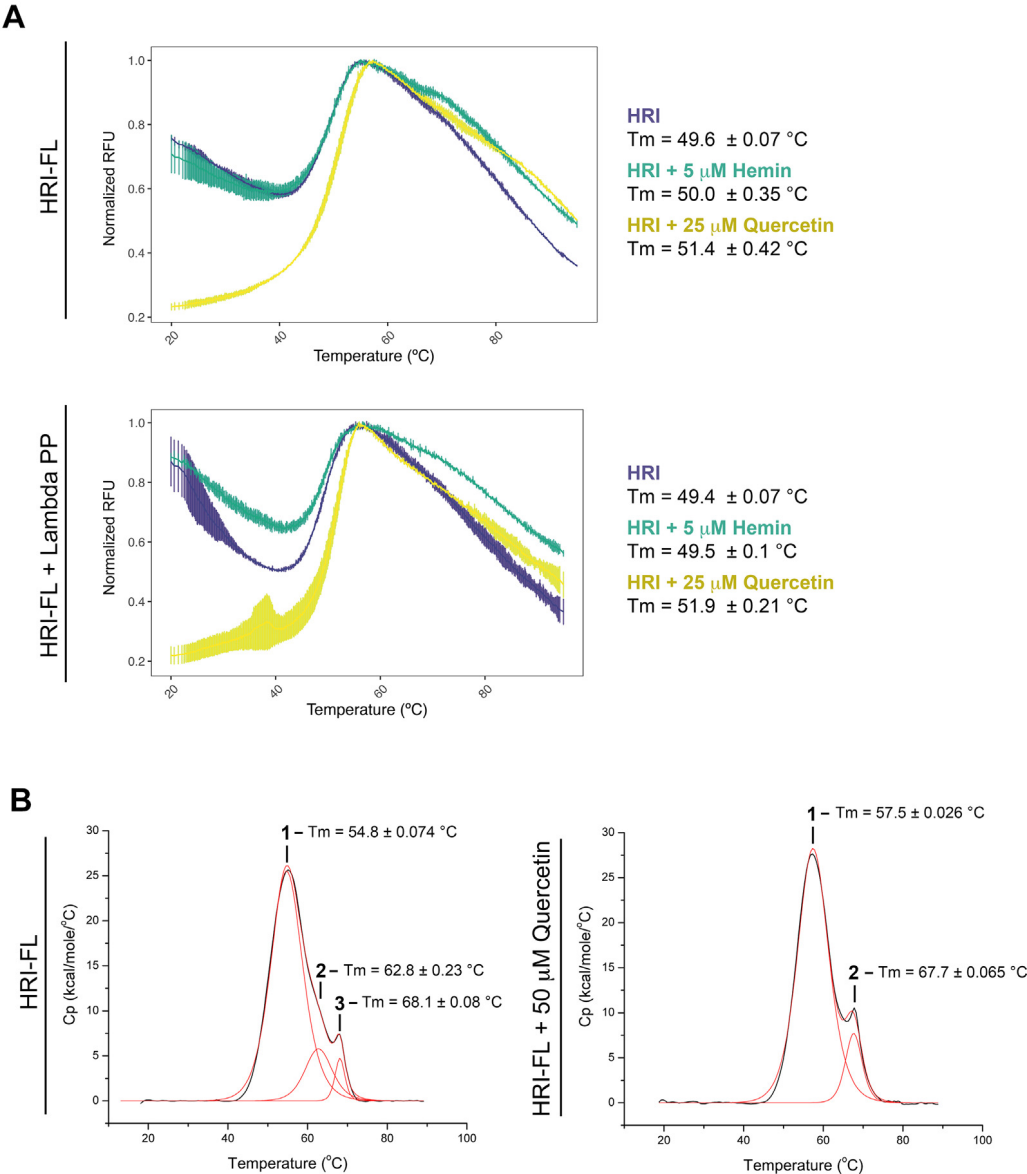
**Analysis of thermostability of HRI-FL in the presence of hemin and quercetin.***A*, DSF analysis of *T*_m_ of HRI-FL and lambda phosphatase–treated HRI-FL in the presence of hemin and quercetin. *B*, DSC analysis of HRI-FL in the presence of quercetin. DSC, differential scanning calorimetry; DSF, differential scanning fluorimetry; HRI, heme-regulated inhibitor; HRI-FL, full-length HRI.

To explore the effect of phosphorylation and hemin binding on protein stability, we employed differential scanning fluorimetry (DSF) studies to assess the *T*_m_. DSF of HRI-FL and dephosphorylated HRI-FL showed a minimal difference in *T*_m_, indicating that one state is not more thermostable than the other (Fig. 4*A*).

*T*_m_ values of HRI-FL and dephosphorylated HRI-FL in the presence of hemin or quercetin was monitored by DSF (Fig. 4*A*). Addition of 25 μM quercetin to 5 μM HRI-FL or dephosphorylated HRI-FL resulted in a ∼2 °C increase in *T*_m_, indicating a mild stabilization upon binding quercetin. No change in *T*_m_ was observed in the presence of 5 μM (greater than sixfold of IC_50_ value) hemin for either HRI-FL or dephosphorylated HRI-FL. It was not feasible to conduct DSF with hemin concentrations above 5 μM because of interference with the fluorescence signal. The thermostability of HRI-FL in the presence of 50 μM quercetin was also monitored by differential scanning calorimetry (Fig. 4*B*). In the absence of quercetin, HRI-FL was observed to have two unfolding peaks with a primary *T*_m_ of 54.8 °C and a shoulder at 62.8 °C. Upon addition of quercetin, one peak was observed with a *T*_m_ of 57.5 °C. Both experiments showed a third peak at ∼68 °C. We have a minor GroEL contaminant in some of our purifications of HRI-FL and propose that this peak represents this contaminant, as it very closely matches published GroEL *T*_m_ values (25). Together, these studies indicate that hemin does not induce a dramatic structural rearrangement of HRI that would drastically alter its thermal stability, whereas quercetin shows thermostabilization of HRI.

### The coiled-coil region of HRI mediates dimer formation

**Figure 5:**
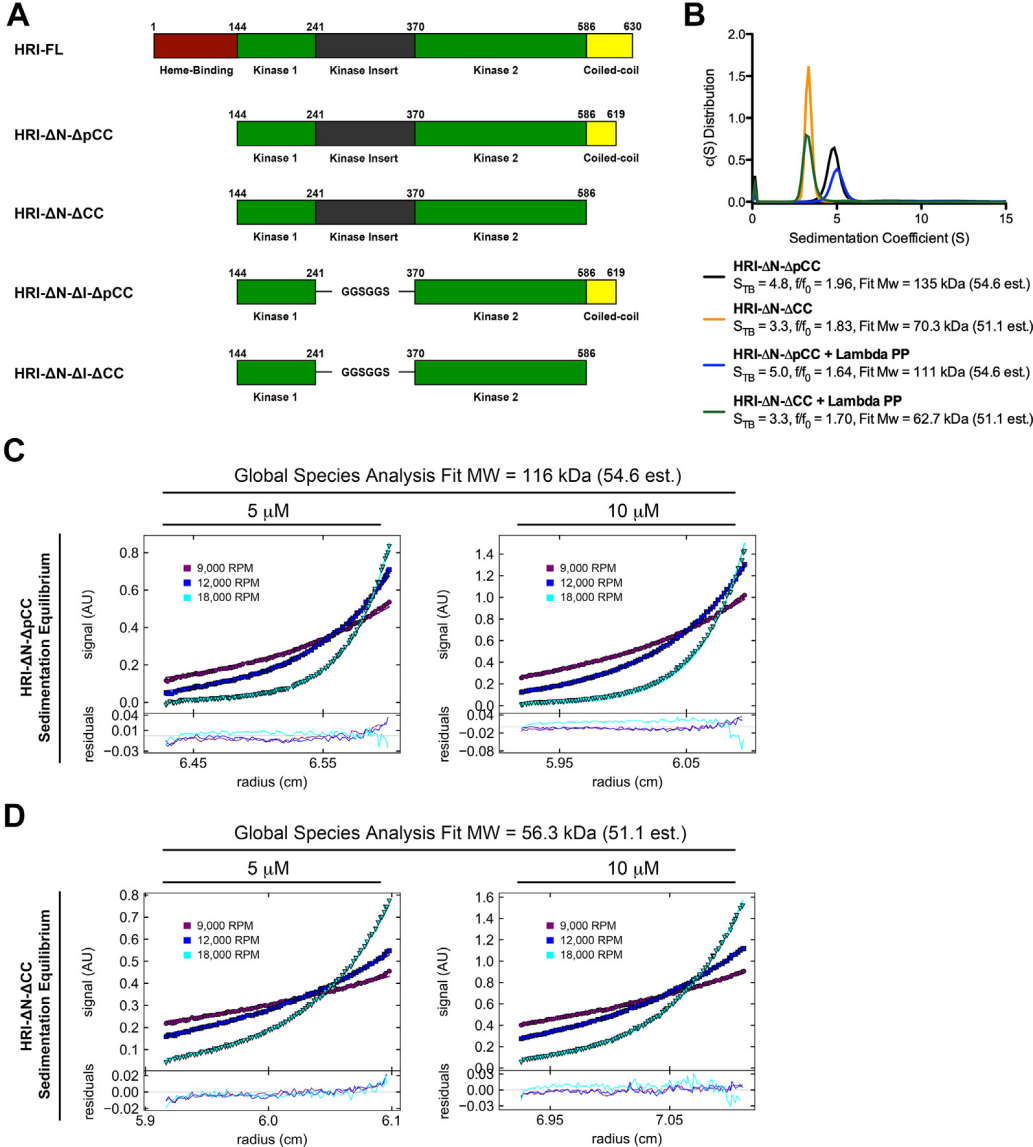
**Domain characterization of HRI.***A*, domain maps of HRI-FL and four engineered domain deletions. *B*, sedimentation velocity analysis of selected phosphorylated and lambda phosphatase–treated HRI constructs. *C*, sedimentation equilibrium analysis of HRI containing partial coiled-coil domain (HRI-ΔN-ΔpCC). *D*, sedimentation equilibrium analysis of HRI missing a complete coiled-coil domain (HRI-ΔN-ΔCC). HRI, heme-regulated inhibitor; HRI-FL, full-length HRI.

To study the functionalities of different domains within HRI, we designed four different truncation and deletion constructs (Fig. 5*A*). HRI-ΔN-ΔpCC removes the N-terminal heme-binding domain (Δ1–143) with partial truncation of the C-terminal coiled-coil domain (Δ620–630). HRI-ΔN-ΔCC removes the N-terminal heme-binding domain with a deletion of the entire coiled-coil domain (Δ587–630). HRI-ΔN-ΔI-ΔpCC represents the HRI-ΔN-ΔpCC construct with a deletion of the kinase insertion sequence within the N-terminal lobe of the kinase domain (Δ242–369). HRI-ΔN-ΔI-ΔCC represents the HRI-ΔN-ΔCC construct with a deletion of the kinase insertion sequence. HRI-ΔN-ΔpCC and HRI-ΔN-ΔCC were purified and subjected to dephosphorylation with lambda phosphatase. Sedimentation velocity analysis was performed on these samples revealing single 4.8 and 5.0 S_T,B_ species for the untreated and dephosphorylated HRI-ΔN-ΔpCC constructs, respectively (Fig. 5*B*). Similar to HRI-FL, the phosphorylated and dephosphorylated HRI HRI-ΔN-ΔpCC constructs displayed different frictional ratios. HRI-ΔN-ΔpCC had an *f*/*f*_0_ = 1.96, whereas dephosphorylated HRI-ΔN-ΔpCC had an *f*/*f*_0_ = 1.64. Both proteins had fit MW values indicative of a dimer species in solution. A single 3.3 S_T,B_ species was observed for untreated and dephosphorylated HRI-ΔN-ΔCC, and the frictional ratios of both proteins showed the same trend of a decreased frictional ratio upon dephosphorylation; indicating a more compact species in solution for the dephosphorylated proteins. Significantly, untreated and dephosphorylated HRI-ΔN-ΔCC fit to MW values indicative of a monomer species in solution, suggesting that the presence of the complete coiled-coil domain is sufficient for HRI dimerization even in the absence of efficient autophosphorylation.

HRI-ΔN-ΔpCC and HRI-ΔN-ΔCC were subjected to sedimentation equilibrium analysis to determine more accurate fits of MW in solution (Fig. 5*C*). Both proteins were analyzed for equilibrium distribution at three centrifugation speeds (9000, 12,000, and 18,000 RPM) and at concentrations of 5 and 10 μM. A global species analysis was implemented in SEDPHAT (sedfitsedphar.nibib.nih.gov) to determine MW. HRI-ΔN-ΔpCC fit to an MW of 116 kDa, indicative of a dimer in solution, whereas HRI-ΔN-ΔCC fit to an MW of 56.3 kDa, indicative of a monomer in solution.

### HRI dimerization facilitates efficient autophosphorylation

**Figure 6:**
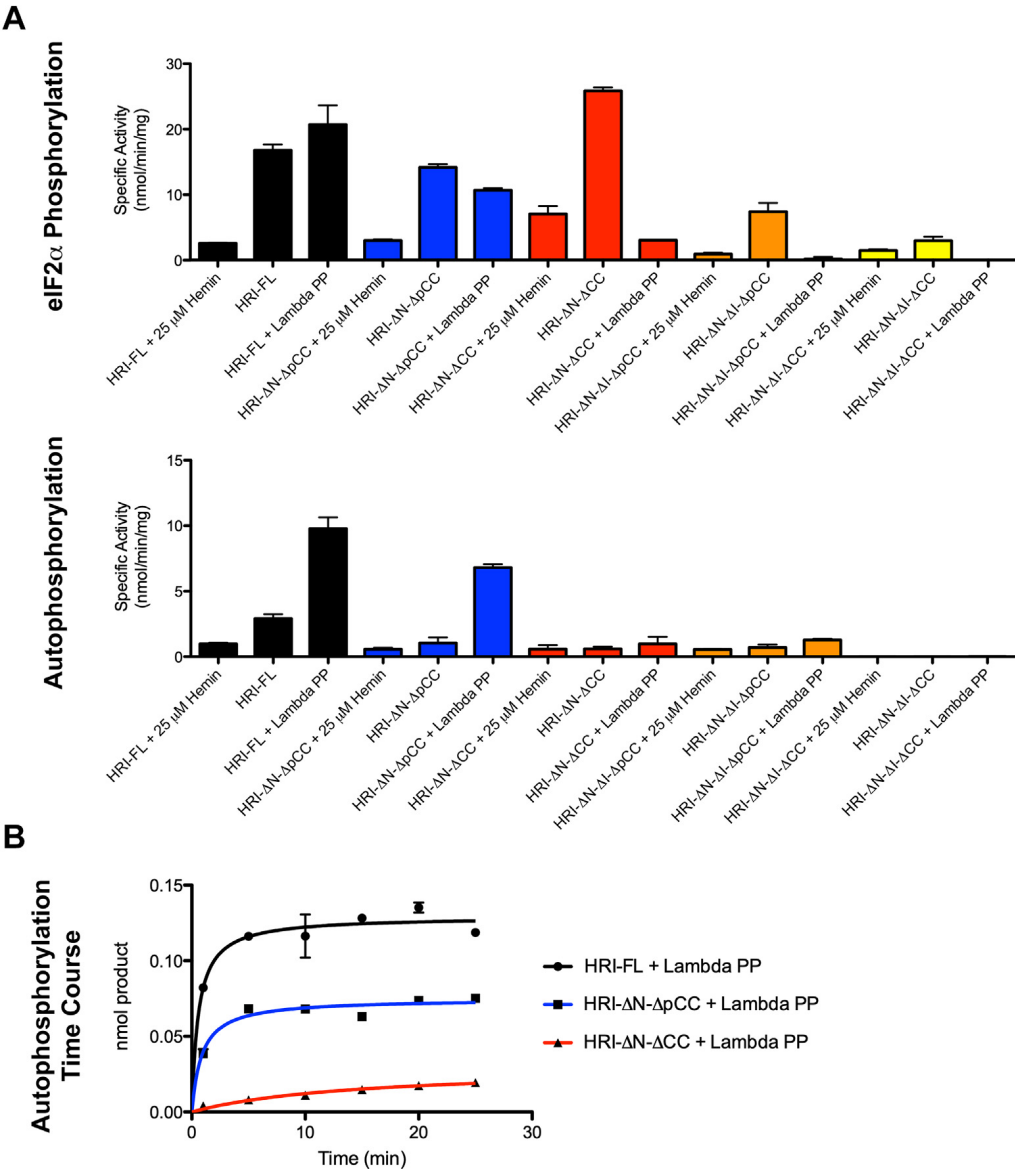
**Analysis of eIF2α peptide phosphorylation and autophosphorylation activity of HRI-FL and domain deletion constructs.***A*, analysis of hemin inhibition and relative eIF2α peptide phosphorylation and autophosphorylation activity for HRI-FL and HRI domain deletion constructs, N = 2. *B*, autophosphorylation time course for HRI deletion constructs. eIF2α, eukaryotic initiation factor 2α; HRI, heme-regulated inhibitor; HRI-FL, full-length HRI.

HRI-FL and the four deletion constructs were studied for effects on eIF2α peptide phosphorylation and autophosphorylation activity (Fig. 6*A*). HRI-FL, HRI-ΔN-ΔpCC, and HRI-ΔN-ΔCC all had comparable levels of eIF2α peptide phosphorylation activity in their phosphorylated states, but upon dephosphorylation, HRI-ΔN-ΔCC was not able to recover activity through autophosphorylation as rapidly as HRI-FL and HRI-ΔN-ΔpCC (Fig. 6*A*). This result suggests that dimerization through the coiled coil facilitates efficient autophosphorylation of HRI.

**Figure 7:**
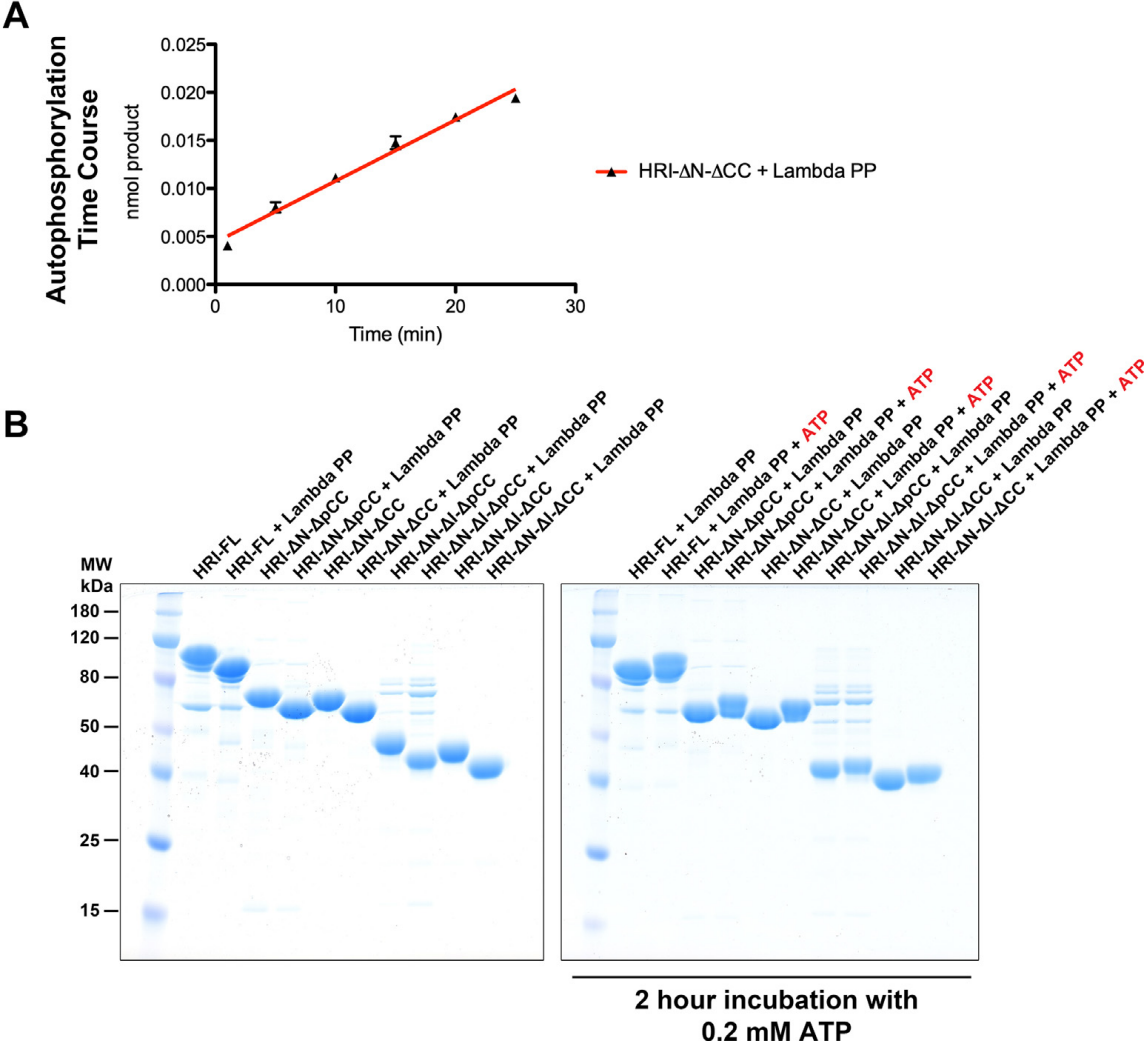
**Time-course autophosphorylation of HRI-FL and domain deletions.***A*, HRI-ΔN-ΔCC autophosphorylation is still linear up to 25 min. *B*, gel shift assay to monitor dephosphorylation and autophosphorylation of HRI-FL and the four domain deletions. HRI, heme-regulated inhibitor; HRI-FL, full-length HRI.

To further elucidate the role of the coiled-coil domain in autophosphorylation, a time course was conducted to monitor HRI-FL, HRI-ΔN-ΔpCC, and HRI-ΔN-ΔCC autophosphorylation activity (Fig. 6*B*). We observed that HRI-ΔN-ΔCC had significantly compromised autophosphorylation activity, suggesting that dimerization facilitates efficient autophosphorylation. HRI-FL and HRI-ΔN-ΔpCC were fully saturated after 5 min, whereas HRI-ΔN-ΔCC was still in the linear range for product formation after 25 min (Fig. 7*A*). To evaluate autophosphorylation over a longer period, we incubated HRI-FL and all four deletion constructs with 200 mM ATP for 2 h and evaluated their autophosphorylation activity by gel shift (Fig. 7*A*). We observed that HRI-ΔN-ΔCC showed a significant level of autophosphorylation after 2 h, indicating that over a long period, it is capable of a moderate level of phosphorylation (either in *cis* or in *trans*). Together, these data demonstrate that HRI dimerization facilitates efficient *trans* autophosphorylation, whereas *cis* autophosphorylation is possible, albeit at a much slower rate.

We also analyzed the phosphorylation activity of the HRI-ΔN-ΔI-ΔpCC and HRI-ΔN-ΔI-ΔCC, which showed compromised eIF2α peptide phosphorylation activity and were unable to efficiently autophosphorylate (Fig. 6*A*). The time-course studies also revealed that these kinase insertion sequence deletion constructs had significantly compromised autophosphorylation activity and were not able to reach their original level of phosphorylation after 2 h (Fig. 7*B*). These results indicate that, while the kinase insertion sequence deletion constructs purify as phosphoproteins from *E. coli* cells, they are significantly slower to autophosphorylate compared with the HRI-FL. Together, these data demonstrate that the kinase insertion sequence is required for both efficient autophosphorylation and maximal eIF2α peptide phosphorylation activities.

### HRI inhibition by hemin occurs primarily through the kinase domain

HRI-FL and the four deletion constructs were also studied for the effects of hemin inhibition and of kinase dephosphorylation on eIF2α peptide phosphorylation and autophosphorylation activities (Fig. 6*A*). We found that addition of 25 μM hemin was able to inhibit HRI-FL and all four deletion constructs. Significantly, hemin was able to inhibit kinase activity with or without an intact N-terminal domain or kinase insert region. These results indicate that heme inhibition of phosphorylation is primarily occurring through a heme-binding site within the kinase domain outside these regions.

## Discussion

Here, we demonstrate that HRI forms a stable dimer in solution that is mediated by the C-terminal coiled-coil domain. We also show that HRI dimerization is independent of the autophosphorylation state, but that efficient autophosphorylation requires dimerization. This suggests a mechanism for HRI transactivation, in which one molecule within the dimer phosphorylates the adjacent molecule, leading to HRI activation. We also investigated the mode of heme regulation of HRI and demonstrate that hemin inhibition of HRI is not directly competing with either ATP or eIF2α peptide substrates and is mediated predominantly by heme binding within the HRI kinase domain.

Previous studies reported wide-ranging oligomerization states for HRI-FL, suggesting it may exist in solution as a dimer, trimer, hexamer, or a larger aggregate (15, 18, 26). Our data demonstrate that HRI-FL purifies from the *E. coli* expression strain as a phosphorylated, active, and stable dimer species. Analytical ultracentrifugation studies reveal that dephosphorylation results in compaction of the shape of the dimer in solution. The more extended conformation of HRI-FL in comparison to dephosphorylated HRI-FL or the catalytically dead HRI-FL-K196M may be partially explained by the activation loop being displaced from the active site upon phosphorylation, a common regulatory mechanism for protein kinases (27, 28). Additional structural studies are necessary to decipher whether loop displacement is the only determinant of species elongation upon autophosphorylation, or if there are other structural rearrangements that contribute to the elongated shape of phosphorylated HRI. Regardless of loop dynamics, these data are consistent with a model where HRI goes through a multistage activation pathway involving an inactive dimer that is activated upon dimer autophosphorylation, consistent with a previous proposal (18).

We demonstrated that HRI is more thermostable in the presence of the inhibitor quercetin. An interesting albeit unsurprising observation is that quercetin has been previously shown to be an ATP-competitive kinase inhibitor, and ATP-competitive compounds have historically been shown to stabilize kinases.

Analysis of HRI domain deletions without the C-terminal coiled-coil domain demonstrates for the first time that the coiled coil is essential for HRI dimer formation. A more elongated species is still observed in phosphorylated HRI with and without the coiled-coil domain, indicating that species elongation is occurring independent of dimerization (Fig. 5*B*). To further enhance our understanding of the HRI coiled-coil domain, the HRI sequence was analyzed with three different coiled-coil prediction programs: Multicoil2 (http://cb.csail.mit.edu/cb/multicoil2/cgi-bin/multicoil2.cgi) (29), Paircoil2 (http://cb.csail.mit.edu/cb/paircoil2/paircoil2.html) (30), and Marcoil (https://toolkit.tuebingen.mpg.de/tools/marcoil) (31) (Table 1). All three programs predict a high-confidence coiled coil with identical register. Multicoil2, the only program to offer oligomer analysis, predicts that dimer formation is more likely than trimer formation for the HRI coiled-coil sequence.

**Table 1:** Coiled-coil analysis of the HRI sequence by three independent algorithms

Program	HRI residue numbers	HRI sequence	Oligomer Probability
		Coiled-coil register	
MultiCoil2	589–619	**VNLTLQMKIIEQEKEIAELKKQLNLLSQDKG**	Dimer > Trimer
		GABCDEFGABCDEFGABCDEFGABCDEFGAB	
PairCoil2	589–620	**VNLTLQMKIIEQEKEIAELKKQLNLLSQDKGV**	NA
		GABCDEFGABCDEFGABCDEFGABCDEFGABC	
Marcoil	590–619	**NLTLQMKIIEQEKEIAELKKQLNLLSQDKG**	NA
		ABCDEFGABCDEFGABCDEFGABCDEFGAB	

Comparison of autophosphorylation and eIF2α peptide phosphorylation activity of HRI-FL and the four domain deletion constructs reveals that HRI-FL and a construct missing the N-terminal domain and part of the C-terminal coiled-coil domain (HRI-ΔN-ΔpCC) had comparable activity toward the eIF2α peptide and are able to efficiently autophosphorylate. These data demonstrate that the N-terminal domain does not play a significant intrinsic role in the autophosphorylation or eIF2α peptide phosphorylation activity of HRI, although other extrinsic factors in cells might influence the regulatory function of the N-terminal domain.

An HRI construct missing the N-terminal and C-terminal coiled-coil domain (HRI-ΔN-ΔCC) purifies from *E. coli* in an active phosphorylated state. Although, upon dephosphorylation, this protein construct is not able to efficiently autophosphorylate to form an activated kinase. These data indicate that dimerization through the coiled-coil domain facilitates efficient autophosphorylation activity, although not an absolute requirement for autophosphorylation as even the coiled-coil deletion constructs slowly accumulate additional phosphates. One HRI molecule within an HRI dimer may have more efficient autophosphorylation activity toward some of the target residues on the adjacent molecule within the same dimer compared with itself. A mechanism of transactivation would be reasonable for HRI as it has been shown to be essential for activation of the related stress kinase PKR (32, 33).

In this study, we also investigated the mode of heme regulation of HRI and demonstrated that hemin binds without apparent cooperativity and does not directly compete with ATP and eIF2α substrates to inhibit HRI, consistent with previously reported mutagenesis data (19). Significantly, hemin inhibited HRI constructs missing the N-terminal heme-binding domain and kinase insert region, suggesting that heme inhibition of phosphorylation is primarily occurring through the heme-binding site within the kinase domain outside the kinase insertion sequence, without significant contribution from the N-terminal heme-binding domain.

With these data, the role of the N-terminal heme-binding domain remains unclear. Previous studies show that interaction between the N-terminal heme-binding domain and the kinase domain is enhanced in the presence of heme and together bind one molecule of heme (19, 24, 34), suggesting that the N-terminal heme-binding domain may structurally contribute to heme inhibition of HRI activity. This idea is supported by data indicating that the interaction between the N-terminal heme-binding domain and kinase domain is stronger when the kinase domain is dephosphorylated (13), suggesting that the N-terminal heme-binding domain may stabilize an inactive confirmation of HRI in the presence of heme. In addition, it has been observed that FL-HRI is more sensitive to heme regulation in comparison to HRI-ΔN when using full-length eIF2α as the substrate for the kinase activity assay (14). It is possible that the N-terminal heme-binding domain may contribute to a structural rearrangement in the presence of heme, leading to a decrease in affinity for the eIF2α substrate. A more rigorous structural analysis of the interdomain interactions of HRI in the presence of heme is warranted to decipher any potential crosstalk between the N-terminal heme-binding domain and the kinase domain of HRI.

**Figure 8:**
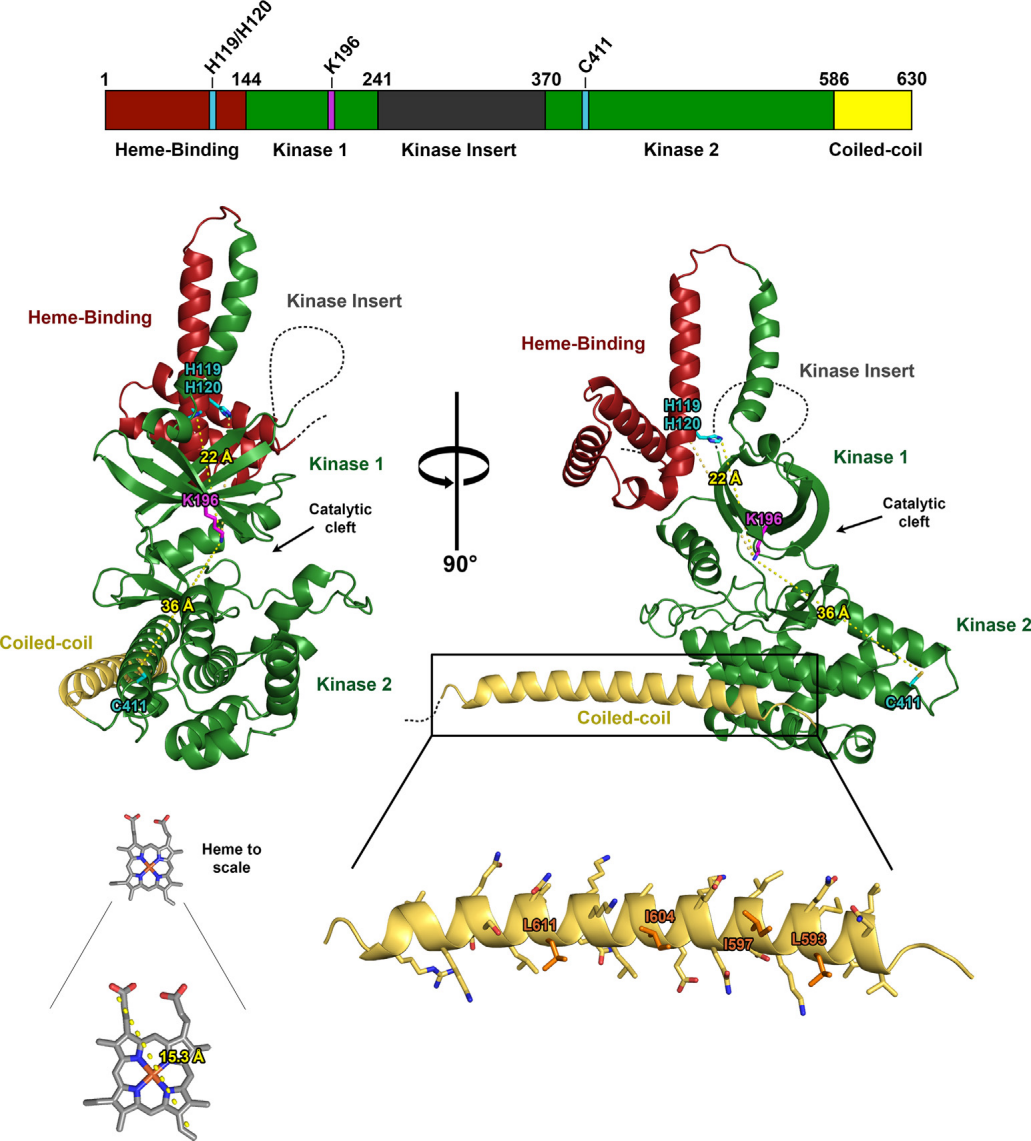
**AlphaFold model of HRI structure.** HRI domain map with relative positions of key heme-chelating and catalytic residues highlighted (*top*). AlphaFold model of HRI three-dimensional structure showing the relative positions of the heme-binding (*red*), kinase (*green*), and coiled-coil (*yellow*) domains and key heme-chelating residues depicted in *cyan* and the catalytic K196 depicted in *magenta*. HRI, heme-regulated inhibitor.

Finally, we employed the recent AlphaFold model of HRI-FL to aid in structural analysis of our biochemical and biophysical data (Fig. 8) (35, 36). In illustrated *cartoon*, unstructured regions are depicted as *dashed lines*, as the AlphaFold model cannot accurately depict the relative positioning of the N-terminal residues of the heme-binding domain (1–60), the kinase insert (241–370), or the disordered residues C terminal to the coiled-coil (625–630). A striking observation is the distance from the catalytic cleft of the residues proposed to function as the heme axial ligands within the heme-binding (H119/H120) and kinase (C411) domains (19). H119 and H120 have been shown to bind heme in a mutually exclusive manner, as mutation of either individually to alanine does not abolish heme binding, but mutation of both does (19). Igarashi *et al*. demonstrated that C411 can also chelate heme, which is consistent with our data indicating that heme inhibition occurs within the kinase domain independent of the N-terminal heme-binding domain. Interestingly, the AlphaFold model predicts that H119/H120 and C411 are quite distant from the ATP-binding site within the catalytic cleft, H119/H120 and C411 measure ∼22 and ∼36 Å from the catalytic lysine, respectively (Fig. 8). These distances, compared with the size of a heme molecule, indicate the unlikelihood of heme directly competing with ATP or substrate within the catalytic cleft in the AlphaFold-predicted orientation of domains.

An additional hypothesis suggests that H119/H120 and C411 may bind to opposing sides of a single molecule of heme through coordination of the heme iron (19). While the AlphaFold model predicts H119/H120 and C411 to be spatially distant, a significant structural reorientation not captured by in this model may represent the true biologically relevant arrangement of regulatory domains. A number of flexible loop regions within the N-terminal heme-binding domain may permit the domain to fold over and access C411 for ternary chelation of heme.

While the AlphaFold model does not capture how dimerization would be mediated through the C-terminal coiled coil, this helix has several hydrophobic residues aligned on one surface of the coil. The residues L593, I597, I604, and L611 occupy the A/D positions within the predicted coiled-coil heptad register and are flanked by charged/polar residues in the E/G positions (Table 1). This arrangement of hydrophobic A/D residues and charged/polar E/G residues is highly indicative of a dimeric coiled coil (37).

Together, these studies highlight the importance of HRI dimerization in autophosphorylation and eIF2α substrate phosphorylation and the heme-binding domain within the kinase domain for HRI heme-sensing activity.

## Experimental procedures

### Generation of expression plasmids

Human HRI (UniProt ID: Q9BQI3; https://www.uniprot.org/uniprot/Q9BQI3) plasmids His-HRI-FL, His-HRI-ΔN-ΔpCC, and His-HRI-ΔN-ΔCC were generated by PCR amplification from HRI-FL complementary DNA (a kind gift from Jeremy Grevet, University of Pennsylvania) (10) and ligated into the EcoRI/XhoI sites of custom-engineered pET28a vector carrying an N-terminal 6xHis tag that is removable by tobacco etch virus protease cleavage. The His-HRI-FL-K196M mutant and Δ242 to 369 deletion constructs (His-HRI-ΔN-ΔI-ΔpCC and His-HRI-ΔN-ΔI-ΔCC) were generated by site-directed mutagenesis (38, 39).

### Protein expression and purification

All constructs were expressed in BL21-Rosetta(DE3) (Novagen). Transformed cells were grown to an absorbance of 0.7 at 600 nm, induced with 0.8 mM IPTG, and expressed overnight at 18 °C. Cells were resuspended in buffer containing 20 mM Tris (pH 8.0), 500 mM NaCl, 5 mM beta-mercaptoethanol, and 20 mM imidazole and lysed with sonication. Lysate was clarified with centrifugation, and supernatant was subjected to nickel affinity chromatography to isolate the His-tagged proteins. Proteins were eluted with 200 mM imidazole. Proteins were then subjected to ion-exchange chromatography using a HiTrap Q column (Cytiva). After ion exchange, proteins were concentrated using a spin concentrator (Millipore). Concentrated proteins were either loaded directly onto a Superdex 200 10/300 (Cytiva) in buffer with 20 mM Hepes (pH 7.5), 150 mM NaCl, and 1 mM Tris(2-carboxyethyl)phosphine (TCEP), or incubated with lambda phosphatase (New England BioLabs) for 1 h at room temperature or 16 h at 4 °C to dephosphorylate prior to loading onto a Superdex 200 10/300 in buffer with 20 mM Hepes (pH 7.5), 150 mM NaCl, and 1 mM TCEP.

### Analytical ultracentrifugation

Sedimentation velocity analysis of all samples was conducted at an absorbance of 0.5 at 280 nm. Experiments were performed with an Optima XL-I analytical ultracentrifuge (Beckman–Coulter) and a TiAn60 rotor with two-channel charcoal-filled epon centerpieces and quartz windows. Data were collected at 20 °C with detection at 280 nm. Absorbance profiles were recorded every 5 min over a period of ∼15 h at 42,000 rpm (141,995*g*). Data were fit using the *c*(*s*) distribution model of the Lamm equation as implemented in SEDFIT (sedfitsedphar.nibib.nih.gov) (40, 41). After optimizing meniscus position and fitting limits, sedimentation coefficient (S_T,B_) and frictional ratio (*f/f*_0_) were determined by interactive least squares fitting of the Lamm equation. For all analyses, the partial specific volume ($\bar{\upsilon }$), solvent density (ρ), and viscosity (η) were derived from chemical composition by SEDNTERP (http://www.rasmb.org/sednterp/) (42).

Sedimentation equilibrium analysis was performed with an Optima XL-I analytical ultracentrifuge. Sedimentation equilibrium samples were loaded into a 6-channel ultracentrifugation cell with buffer blanks. Equilibrium distribution data were collected at 4 °C using a TiAn60 rotor at speeds of 9000, 12,000, and 18,000 RPM. Species analyses were carried out with global fits to data acquired at multiple speeds for each concentration using the program SEDPHAT (43).

### Activity assay

HRI activity was monitored using a filter paper–based γ^32^P ATP assay (44). An eIF2α peptide (Genscript) was employed as a substrate. The peptide contains residues 46 to 57 (ILLSELSRRRIRRWGRPVGRRRRP) of eIF2α and was engineered with a positively charged C-terminal sequence to enhance binding to the negatively charged P81 filter paper (St Vincent’s Institute of Medical Research). Phosphorylation reactions were carried out at room temperature in a buffer containing 20 mM Hepes (pH 7.5), 150 mM NaCl, 5 mM MgCl_2_, and 1 mM TCEP. Reactions were initiated with the addition of an ATP solution supplemented with γ^32^P ATP (PerkinElmer). All data points were collected in duplicate, and specific activity was calculated using a γ^32^P ATP standard curve generated with each experiment.

HRI bound to the p81 paper in addition to the eIF2α peptide, leading to autophosphorylation background activity. Therefore, for each peptide phosphorylation data point, an autophosphorylation control data point lacking peptide was also collected. Peptide phosphorylation data were generated by subtracting the autophosphorylation signal from the total phosphorylation signal of the peptide-containing reaction. Steady-state kinetic analysis of HRI-FL was used to determine the saturating fixed concentrations of ATP (200 μM) and eIF2a peptide (200 μM) used in the IC_50_, competition, and HRI truncation/deletion activity experiments. Hemin (Sigma–Aldrich) was resuspended in 0.01 M NaOH to make a 2.5 mM stock solution. Quercetin (Sigma–Aldrich) was resuspended in dimethyl sulfoxide to make a 10 mM stock solution. All data were fit using GraphPad Prism, version 5.0. Steady-state data were fit in using the Michaelis–Menten and substrate inhibition equations. IC_50_ data were fit using the log(inhibitor) *versus* response − variable slope equation.

### DSF

Proteins were incubated with 5 × SYPRO Orange (Thermo Fisher Scientific) and subjected to denaturation over a temperature gradient (45). Fluorescence at 570 nm was monitored from 20 to 95 °C using a TAQMAN 7900 QPCR (Life Technologies). *T*_m_ values were estimated, and figures were generated using the DSF world server. Addition of hemin strongly decreased the total fluorescence signal; data were normalized to one for ease of comparison.

## Data availability

All data contained within the article are available upon request from the corresponding author.

## Conflict of interest

The authors declare that they have no conflicts of interest with the contents of this article.
